# Beneficial Effect of 7-*O*-Galloyl-D-sedoheptulose, a Polyphenol Isolated from Corni Fructus, against Diabetes-Induced Alterations in Kidney and Adipose Tissue of Type 2 Diabetic *db/db* Mice

**DOI:** 10.1155/2013/736856

**Published:** 2013-11-20

**Authors:** Chan Hum Park, Jeong Sook Noh, Jong Cheol Park, Takako Yokozawa

**Affiliations:** ^1^College of Pharmacy, Pusan National University, Busan 609-735, Republic of Korea; ^2^Department of Food Science & Nutrition, Pusan National University, Busan 609-735, Republic of Korea; ^3^Department of Oriental Medicine Resources, Sunchon National University, Jeonnam 540-742, Republic of Korea; ^4^Graduate School of Science and Engineering for Research, University of Toyama, Toyama 930-8555, Japan; ^5^Molecular Inflammation Research Center for Aging Intervention, Pusan National University, Busan 609-735, Republic of Korea

## Abstract

Traditional medicines are being focused on as possible treatments for diabetes and its complications because of their negligible toxic and/or side effects. In line with this, our group has reported that Corni Fructus, a traditional medicine considered exhibiting beneficial effects on liver and kidney functions, possessed an antidiabetic effect *via* ameliorating glucose-mediated metabolic disorders. To add to these findings, we screened the iridoid glycoside fraction containing morroniside and loganin, and low molecular weight polyphenol fraction containing 7-*O*-galloyl-d-sedoheptulose (GS) from Corni Fructus. To our knowledge, GS is a compound only detected in Corni Fructus, and its biological activity has been poorly understood until now. For these reasons, we examined whether GS has an ameliorative effect on diabetic changes using type 2 diabetic *db/db* mice. Our findings suggest that GS has a beneficial effect on the pathological state of the serum, kidney, and adipose tissue related to diabetic damage.

## 1. Background

Diabetes is a metabolic disorder known to cause deleterious changes in various tissues exhibited as diabetic complications triggered by hyperglycemia, dyslipidemia, oxidative stress, inflammation, and advanced glycation [1]. Among these pathogenic factors in diabetes, abnormal lipid metabolism and hyperglycemia-induced oxidative and carbonyl stress (so-called lipotoxicity and glucotoxicity) play a central role in the initiation and progression of diabetes-related disease [2]. Chronic hyperglycemia and dyslipidemia cause oxidative stress and inflammatory responses through the formation of advanced glycation end-products (AGEs) [3, 4], activation of the protein kinase C pathway [5, 6], increased glucose flux through the polyol pathway [7], and the accelerated generation of reactive oxygen species (ROS) [8, 9]. The resulting glycative, glycoxidative, and carbonyl lipotoxicity and oxidative stresses play a key role in the pathogenesis of diabetes [10–13]. Therefore, the attenuation of oxidative stress and regulation of hyperlipidemia have been considered as ways to alleviate diabetes and diabetic complications.

Clinical evidence has suggested that the appropriate use of traditional Chinese medicines with modern Western medicinal, or mainstream antidiabetic drugs, can prevent or ameliorate the development of diabetic complications. Many diabetic patients choose alternative therapeutic approaches such as herbal or traditional Chinese medicine along with mainstream antidiabetic drugs, thus making alternative therapy for diabetes very popular [14]. However, these medicines usually have an insufficient scientific basis, and the exact mechanisms behind their beneficial effects are unknown. Therefore, recently, based on a large number of chemical and pharmacological studies, numerous bioactive compounds have been identified in Chinese medicinal plants for diabetes [15], and we have investigated the mechanism and bioactive constituents of Corni Fructus, the fruit of *Cornus officinalis* Sieb. et Zucc. (Cornaceous), in diabetic animal models.

Corni Fructus is an important crude herb used in Chinese medicine. It is considered to be one of the 25 plant-based drugs most frequently used in China, Japan, and Korea. It is known to exhibit several biological activities, including hypoglycemic, antineoplastic, and antimicrobial effects, and improve liver and kidney functions [16–18]. We previously reported that treatment with Corni Fructus for 10 days suppressed hyperglycemia, proteinuria, renal AGE formation, and related protein expressions, that is, receptor for AGEs (RAGE), nuclear factor-kappa B (NF-*κ*B), transforming growth factor-*β*_1_ (TGF-*β*_1_), and *N* ^*ε*^-(carboxymethyl)lysine (CML), in the same way as with aminoguanidine. However, improvement of the renal function, shown *via* serum creatinine and creatinine clearance, was superior to aminoguanidine treatment [19]. In addition, the administration of Corni Fructus inhibited the elevation of both systolic and diastolic blood pressures, and lowered serum total cholesterol levels with a decrease in esterified cholesterol in a diet-induced hypercholesterolemia rat model [20]. Moreover, the atherogenic index was decreased in a dose-dependent manner, suggesting its protective role against cardiovascular disease through regulating cholesterol and lipoprotein levels [20]. Therefore, Corni Fructus was suggested to have beneficial effects on diabetes and diabetic complications.

**Figure 1:**
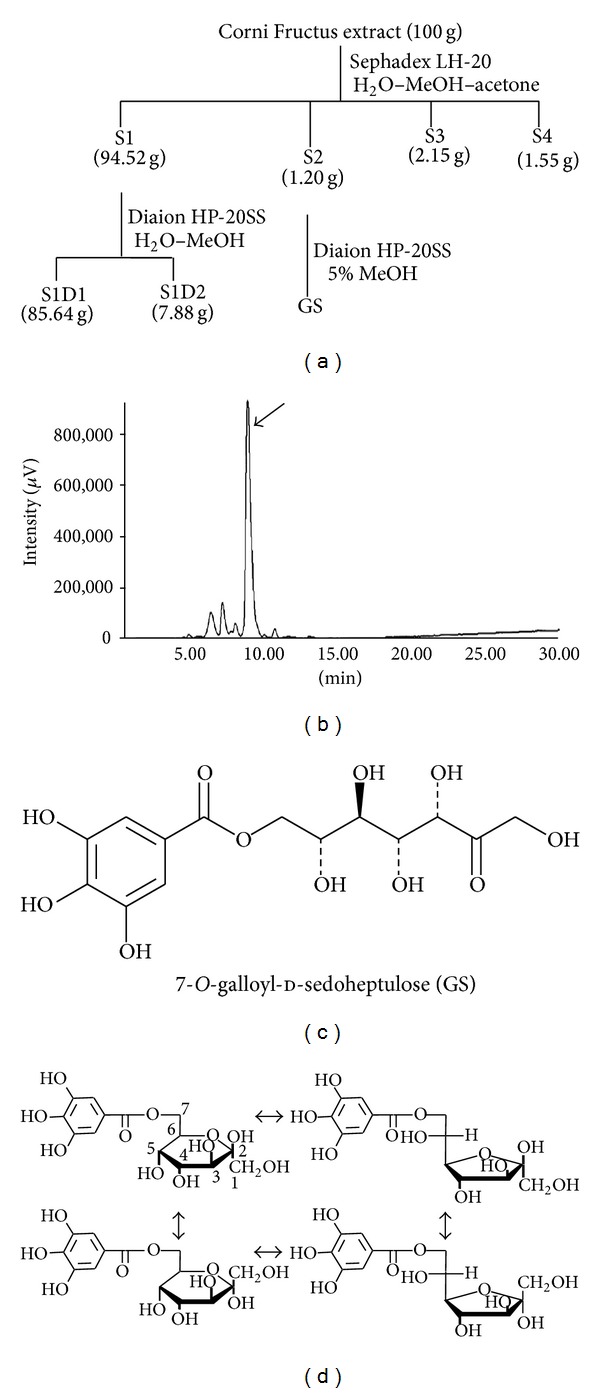
Fractionation of Corni Fructus, HPLC profile of GS, and its structure. (a) Fractionation of Corni Fructus was performed as described in* Biological & Pharmaceutical Bulletin*, vol. 30, no. 7, pp. 1289–1296, 2007. (b) HPLC profile. The large peak shown by the arrow is the structure of GS, as described in (c), and the other peaks represent its four isomers, as described in (d).

The discovery of efficacious components is essential for clarification of the precise mechanisms of herbal medicines. However, studies on the biological activities of the active components in Corni Fructus are limited. Therefore, we isolated the major active components of Corni Fructus by employing activity-guided fractionation (Figure 1), and the effects of morroniside, loganin, and 7-*O*-galloyl-d-sedoheptulose (GS) were assessed on glucose metabolism, AGE formation, oxidative stress, and inflammation in type 2 diabetic liver, kidney, pancreas, and adipose tissue to identify their effects and mechanism of action in type 2 diabetes [21–30]. Among the isolated components of Corni Fructus which were suggested to be important contributors to prevent and/or delay the onset of diabetic disease, GS, to our knowledge, is a compound only detected in Corni Fructus [31]. Part of the sugar (sedoheptulose) in GS is ketoheptose, a monosaccharide with seven carbon atoms and a ketone functional group. Sedoheptulose is a seven-carbon ketose sugar originally found in *Sedum spectabile*, a common perennial garden plant. It is often a part of the human diet. This sugar, d-sedoheptulose (I), is a significant intermediary compound in the cyclic regeneration of d-ribulose. It also plays an important role as a transitory compound in the cyclic regeneration of d-ribulose for carbon dioxide fixation in plant photosynthesis.

Therefore, this paper presents a review of our recent findings, with emphasis on the therapeutic potential of the polyphenol, GS, of Corni Fructus against diabetic damage in the kidney and adipose tissue.

## 2. Type 2 Diabetic *db/db* Mice

To investigate the effect of GS, *db/db* mice were used. A spontaneous mutant strain, C57BLKS/J *db/db* mice, has the *db* mutation, a splicing mutation caused by a point mutation in the downstream intron of the leptin receptor gene, and so it is unresponsive to leptin. Leptin is a peptide hormone secreted by adipocytes and is involved in eating behavior and energy homeostasis. For this reason, after birth, the homozygous diabetic (*db/db*) mice show unrepressed eating behavior, become obese, and develop severe insulin resistance associated with hyperinsulinemia and hyperglycemia [32]. In this study, *db/db* mice showed diabetic characteristics, such as hyperglycemia, hyperleptinemia, and hyperinsulinemia, compared with homozygous control (*m/m*) mice, as presented in Table 1. GS administration significantly reduced serum leptin and insulin levels at a dose of 100 mg/kg, while the serum glucose level was slightly decreased without significance. The serum C-peptide level was compared as an indirect biomarker of insulin secretion. As expected, there was a significant increase in the serum C-peptide level in the vehicle-treated *db/db* group, which was closely associated with the increased removal of blood glucose (Table 1). Thus, GS treatment prevents diabetes in *db/db* mice, as evidenced by improved insulin sensitivity through the maintenance of normal insulin and glucose levels and the preservation of insulin and C-peptide levels in the serum, meaning that GS can ameliorate impaired glucose and insulin tolerance in *db*/*db* mice.

## 3. GS Ameliorates Renal Damage Triggered by ROS-Sensitive Pathway of Inflammation and Apoptosis

Initial diabetic renal damage is known to involve hyperglycemia-induced oxidative stress. Increased oxygen and peroxy radicals aggravate tissue oxidative stress, which affects the oxidation of important macromolecules including proteins, lipids, carbohydrates, and DNA chains. Moreover, ROS activates the signal transduction cascade and transcription factors and overexpression of genes and proteins in glomerular mesangial and tubular epithelial cells, leading to pathological changes in the kidney [33]. Therefore, in this study, we investigated the effect of GS on the oxidative stress and ROS-related factors involved in the development of diabetic renal damage using type 2 diabetic C57BLKS/J *db/db* mice.

**Figure 2:**
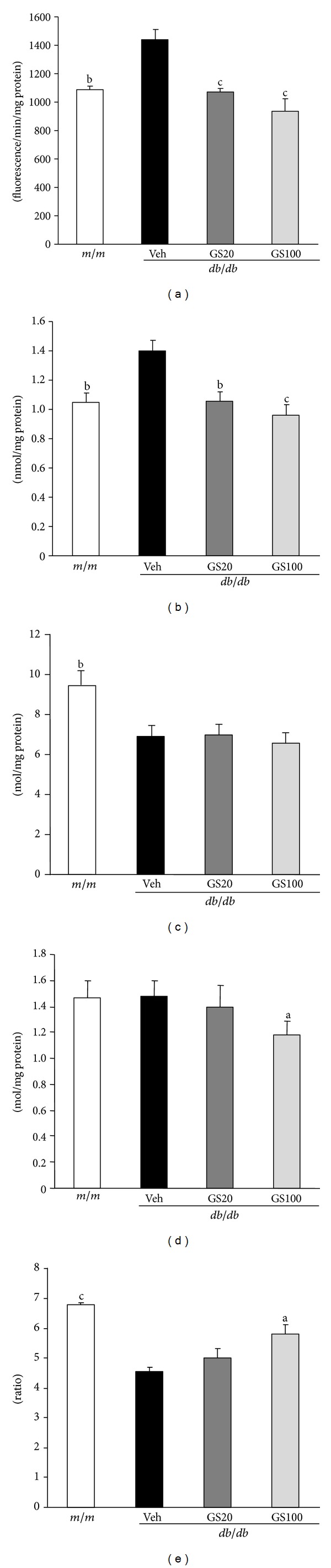
ROS (a), TBARS (b), GSH (c), GSSG (d), and GSH/GSSG (e) levels in the kidney. *m/m*, Misty; Veh, vehicle-treated *db/db* mice; GS20, GS 20 mg/kg body weight-treated* db/db* mice; GS100, GS 100 mg/kg body weight-treated* db/db* mice. The results are presented as the means ± S.E.M. ^a^ *P* < 0.05, ^b^ *P* < 0.01 versus vehicle-treated *db/db* mouse values.

As shown in Figure 2, GS effectively attenuated oxidative stress *via* a decrease in ROS and thiobarbituric acid-reactive substance (TBARS) levels as well as an enhanced reduced glutathione (GSH)/oxidized glutathione (GSSG) ratio. In addition, increased serum urea nitrogen and creatinine levels associated with an abnormal renal function were significantly lowered by GS treatment.

**Figure 3:**
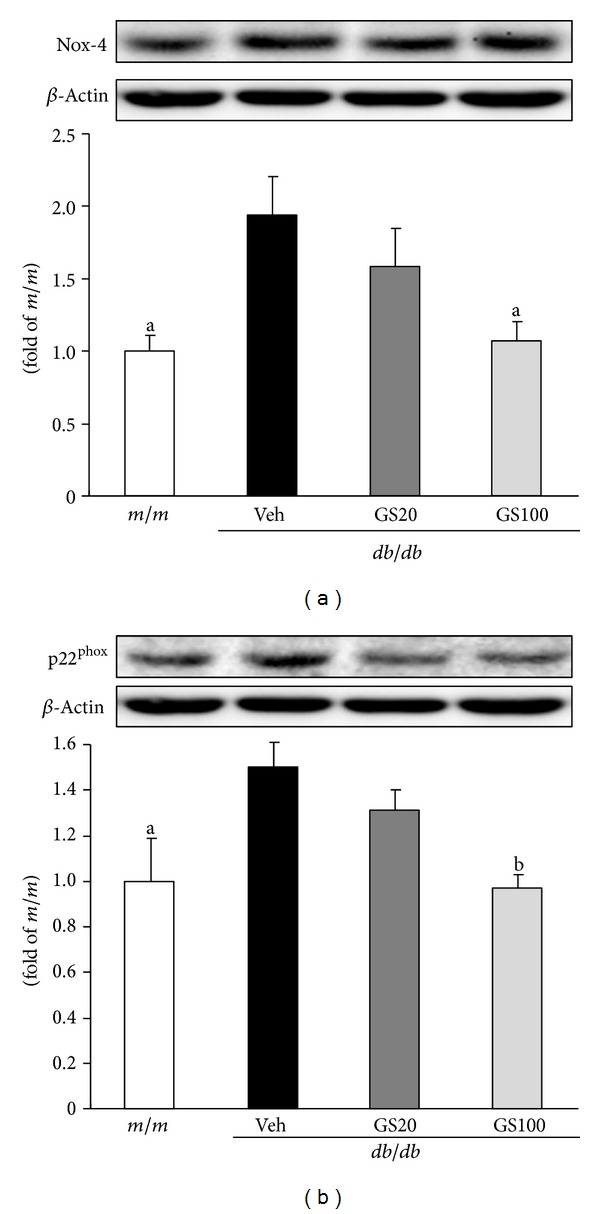
Nox-4 (a) and p22^phox^ (b) protein expressions in the kidney. *m/m*, Misty; Veh, vehicle-treated *db/db* mice; GS20, GS 20 mg/kg body weight-treated* db/db* mice; GS100, GS 100 mg/kg body weight-treated* db/db* mice. The results are presented as the means ± S.E.M. ^a^ *P* < 0.05, ^b^ *P* < 0.01 versus vehicle-treated *db/db* mouse values.

In the diabetic kidney, enzymatic and nonenzymatic sources of ROS include autoxidation of glucose, transition metal-catalyzed Fenton reactions, advanced glycation, polyol pathway flux, mitochondrial respiratory chain deficiencies, and nicotinamide adenine dinucleotide phosphate (NADPH) oxidase [34]. Although the origin of increased ROS generation in renal disease is multifactorial, recent studies have focused on the fact that NADPH oxidase mainly participates in the process of ROS generation [35–37]. There is accumulating evidence that nonphagocytic NADPH oxidases are major enzymatic sources of ROS generation in ischemia-reperfusion injury, inflammation, hypertension, and atherosclerosis based on experimental animal and human studies [38, 39]. Also, renal NADPH oxidase expression was reported to be enhanced in glomeruli and distal tubules in the presence of diabetic nephropathy [40]. Structurally, NADPH oxidase comprises the membrane-associated cytochrome *b*_558_, composed of one p22^phox^ and one gp91^phox^ subunit and at least four cytosolic subunits (p47^phox^, p67^phox^, p40^phox^, and the small GTP_ase_ *rac*1 or *rac*2) [41]. In particular, Nox-4 and p22^phox^ were found to be a major source of ROS production in the kidney and could play a role in pathological conditions [35, 42, 43]. Also, in a rodent model of type 2 diabetes (*db/db* mouse), the renal expression of Nox-4 and p22^phox^ was increased, and this was associated with ROS-induced renal damage [44]. Therefore, we examined the renal protein expression of Nox-4 and p22^phox^, subunits of NADPH oxidase, to identify the exact mechanism behind the reduction of renal ROS levels in the GS-treated group. In Western blot analysis, Nox-4 and p22^phox^ protein expressions were significantly upregulated in the type 2 diabetic kidney; however, GS 100 mg/kg administration significantly normalized the increased subunits of NADPH oxidase (Figure 3). These results indicate that the inhibitory effect of GS on ROS generation was due to the downregulated expression of NADPH oxidase in *db/db* mice.

**Figure 4:**
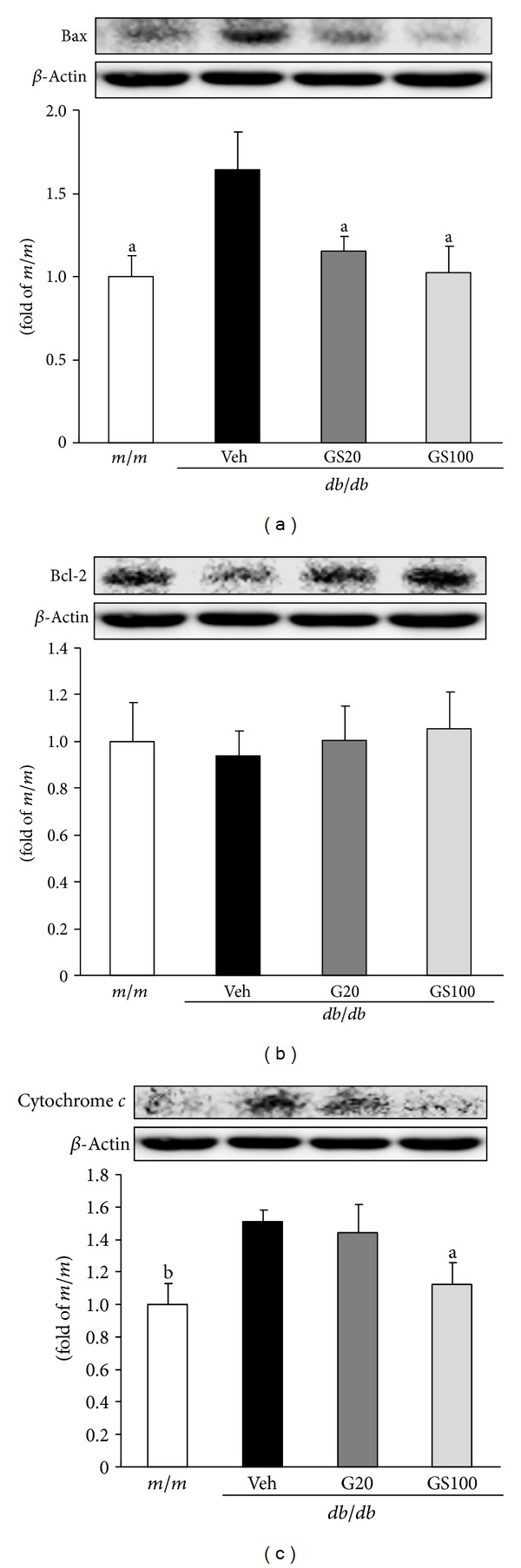
Bax (a), Bcl-2 (b), and cytochrome *c* (c) protein expressions in the kidney. *m/m*, Misty; Veh, vehicle-treated *db/db* mice; GS20, GS 20 mg/kg body weight-treated* db/db* mice; GS100, GS 100 mg/kg body weight-treated* db/db* mice. The results are presented as the means ± S.E.M. ^a^ *P* < 0.05, ^b^ *P* < 0.01 versus vehicle-treated *db/db* mouse values.

Furthermore, ROS has been shown to induce apoptosis in the proximal tubular cells of an animal model of unilateral ureteral obstruction [9]. Apoptotic cells have been detected in both proximal and distal tubular epithelia of human and experimental diabetic kidneys [45], suggesting that apoptosis is also involved in the loss of tubular cells in diabetic nephropathy. Increased mitochondrial superoxide production initiates a range of damaging reactions through the production of H_2_O_2_, ferrous iron, ^*∙*^OH, and ONOO^−^, which can then damage lipids, proteins, and nucleic acids. A number of functional enzymes within the mitochondria are particularly susceptible to ROS-mediated damage, leading to altered ATP synthesis, cellular calcium dysregulation, and the induction of mitochondrial permeability transition, all of which predispose the cell to necrosis or apoptosis. Podocyte apoptosis has been proposed as a new cellular pathomechanism in diabetic nephropathy [46]. Apoptosis is most likely caused by changing the balance in the expression of the anti- and proapoptotic molecules, Bcl-2 and Bax, respectively. While Bcl-2 expression may account for the maintenance of glomerular hypercellularity, Bax expression might be more important in cell loss leading to glomerulosclerosis. Bax forms oligomers, thereby increasing mitochondrial permeability and facilitating the release of cytochrome *c* from the mitochondrial intermembrane space. Once released from the mitochondria, cytochrome *c* further activates apoptosis. In this study, GS administration in *db/db* mice significantly suppressed renal protein expression of Bax and cytochrome *c*, although there was no change in Bcl-2 protein levels among all experimental groups (Figure 4). These results suggest that GS prevents apoptosis-induced renal damage, at least in part, through the amelioration of oxidative stress-induced mitochondrial dysfunction.

**Figure 5:**
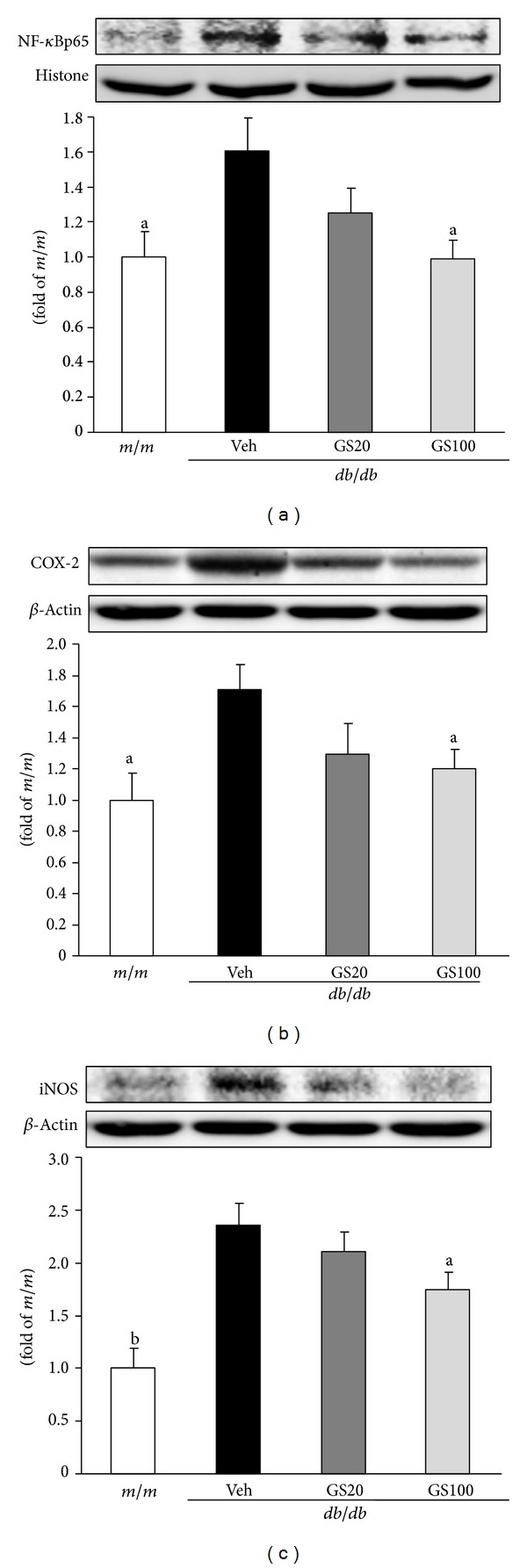
NF-*κ*Bp65 (a), COX-2 (b), and iNOS (c) protein expressions in the kidney. *m/m*, Misty; Veh, vehicle-treated *db/db* mice; GS20, GS 20 mg/kg body weight-treated* db/db* mice; GS100, GS 100 mg/kg body weight-treated* db/db* mice. The results are presented as the means ± S.E.M.   ^a^ *P* < 0.05, ^b^ *P* < 0.01 versus vehicle-treated *db/db* mouse values.

On the other hand, NF-*κ*B is one of the crosstalk points of multiple signal transduction pathways, and plays a key role in the regulation of transcription and expression of many genes involved in inflammatory responses [47, 48]. For example, enhanced oxidative stress leads to NF-*κ*B transcription and, consequently, induces expressions of its related proinflammatory factors such as cyclooxygenase-2 (COX-2) and inducible nitric oxide synthase (iNOS) [49]. In humans, COX-2 expression is readily detectable in glomerular podocytes of adults [50, 51], and its expression level has been reported to increase during acute renal allograft rejection [52, 53]. In cultured podocytes, COX-2 overexpression led to more marked cytoskeletal disorganization and apoptosis in response to high-glucose stimulation [54]. These changes were ameliorated by treatment with a specific COX-2 inhibitor, indicating that podocyte COX-2 expression increases susceptibility to the development of diabetic nephropathy [54]. Meanwhile, the rapid induction of iNOS expression can trigger NO-dependent apoptosis *in vitro*, which appears to result from DNA damage and may be mediated by a p53-dependent apoptotic pathway [55]. iNOS expression is typically absent in unstimulated cells, but is markedly induced by proinflammatory cytokines including tumor necrosis factor-*α* (TNF-*α*), interleukin (IL)-1, and IL-6 [56, 57]. For that reason, proinflammatory factors such as NF-*κ*B and its transcriptional factors have been important target genes to prevent further renal damage caused by the inflammatory response and apoptosis. In this study, GS administration to type 2 diabetic *db/db* mice caused significant renal protein downregulation of NF-*κ*B, COX-2, and iNOS (Figure 5), suggesting that GS efficiently inhibited renal inflammation-related injury in *db/db* mice.

**Figure 6:**
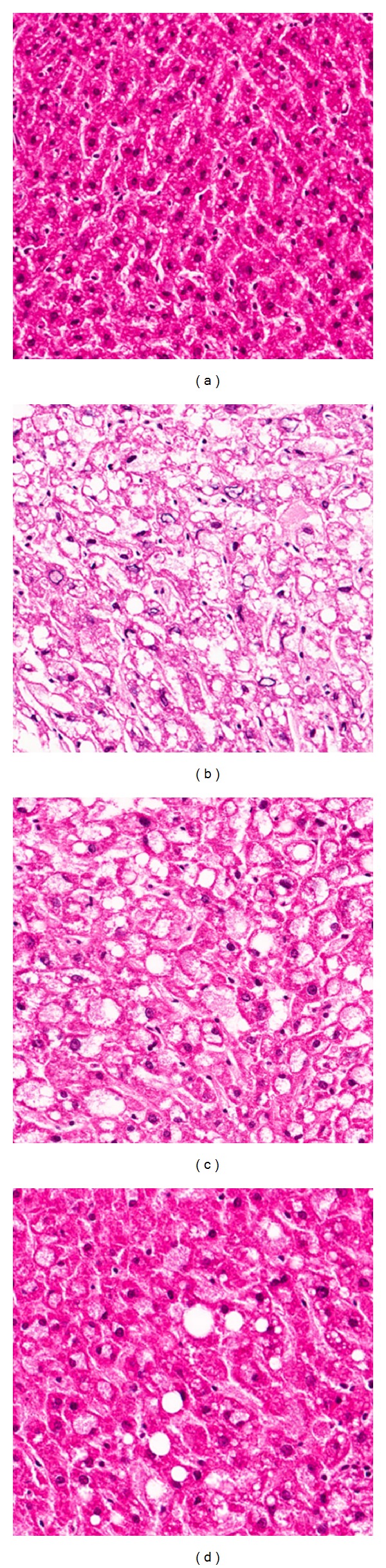
HE staining of the kidney. (a) Misty, (b) vehicle-treated *db/db* mice, (c) GS 20 mg/kg body weight-treated* db/db* mice, and (d) GS 100 mg/kg body weight-treated* db/db* mice. ×200.

This study supports the concept that, in hyperglycemia, enhanced oxidative stress, upregulation of NADPH oxidase and apoptosis, and NF-*κ*B-related inflammation are associated with renal damage in type 2 diabetes. GS administration effectively alleviated these unfavorable responses in the presence of diabetic injury of kidney, as shown in Figure 6. Therefore, this study suggests that GS exerts its renal protective potential through the inhibition of oxidative stress-sensitive mechanisms of apoptosis and the proinflammatory response in the kidney of type 2 diabetics.

## 4. GS Acts as a Regulator of Oxidative Stress, Inflammation, and Fibrosis in Adipose Tissue

Adipose tissue stores energy in the form of lipids and releases fatty acids in response to nutritional signals or energy insufficiency. In addition, adipocytes have endocrine functions, secreting hormones and factors that regulate physiological functions such as the immune response, insulin sensitivity, and food intake [58]. Excessive fat accumulation in the body and white adipose tissue causes obesity and results in an increased risk of many serious diseases, including type 2 diabetes, hypertension, and heart disease. In the present study, we examined whether GS could prevent the gluco- and lipotoxicity of adipose tissue triggered by the ROS-sensitive pathway of inflammation and fibrosis in type 2 diabetic *db/db* mice.

**Figure 7:**
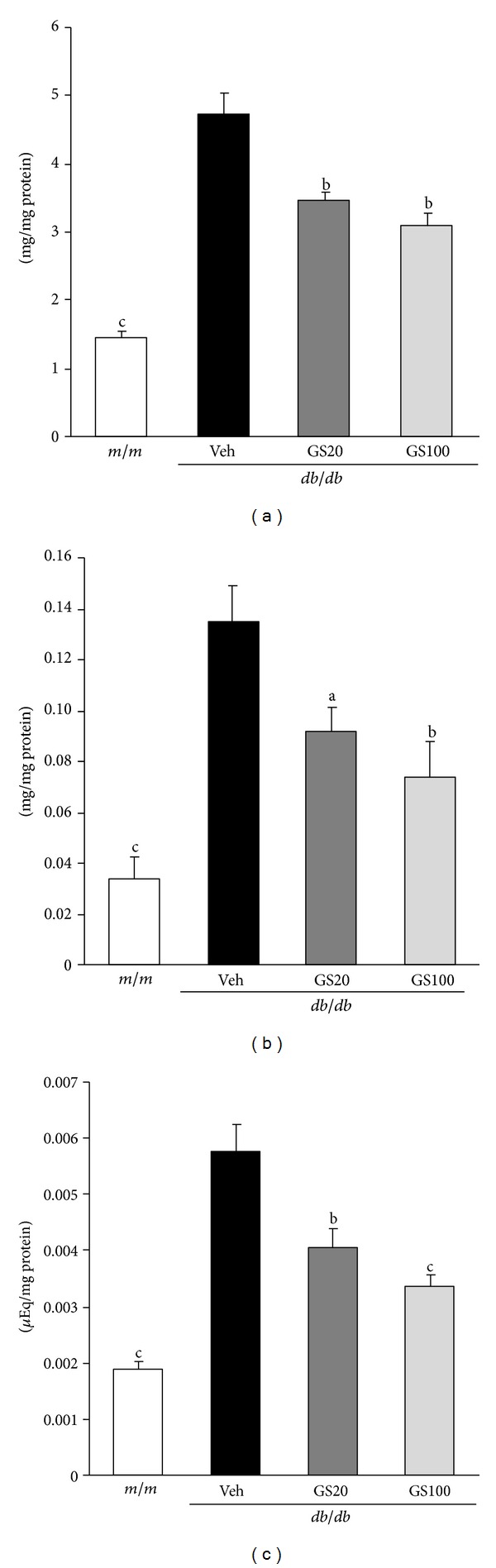
Triglycerides (a), total cholesterol (b), and NEFA (c) contents in the adipose tissue. *m/m*, Misty; Veh, vehicle-treated *db/db* mice; GS20, GS 20 mg/kg body weight-treated* db/db* mice; GS100, GS 100 mg/kg body weight-treated* db/db* mice. The results are presented as the means ± S.E.M.  ^a^ *P* < 0.05, ^b^ *P* < 0.01, ^c^ *P* < 0.001 versus vehicle-treated *db/db* mouse values.

**Figure 8:**
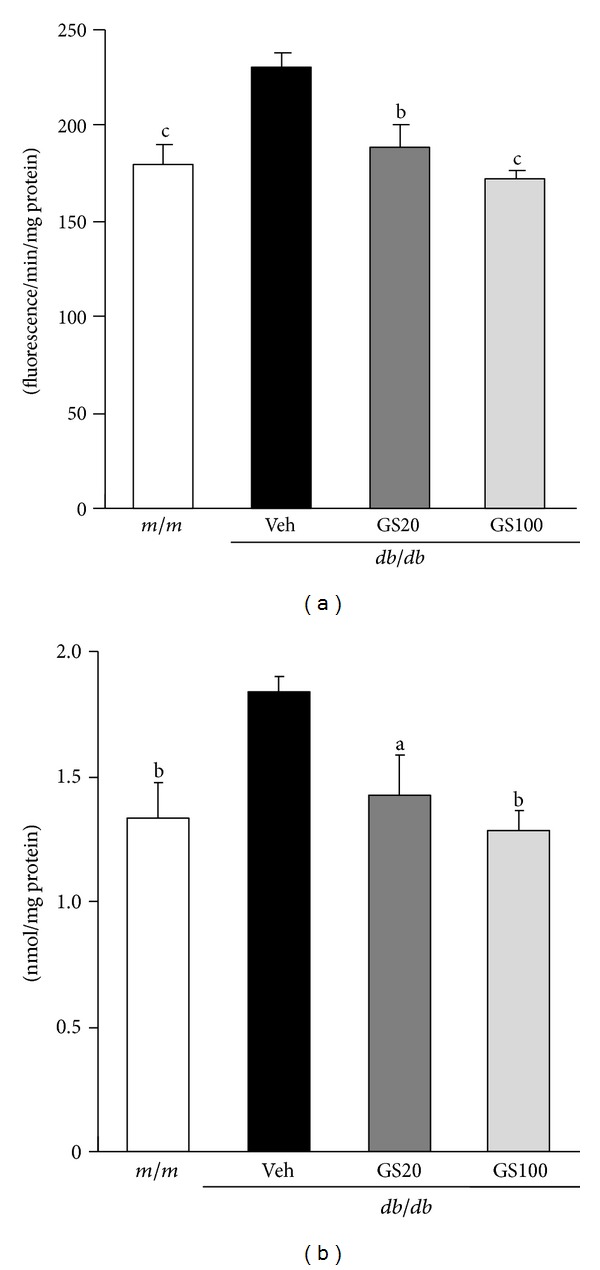
ROS (a) and TBARS (b) levels in the adipose tissue. *m/m*, Misty; Veh, vehicle-treated *db/db* mice; GS20, GS 20 mg/kg body weight-treated* db/db* mice; GS100, GS 100 mg/kg body weight-treated* db/db* mice. The results are presented as the means ± S.E.M. ^a^ *P* < 0.05, ^b^ *P* < 0.01, ^c^ *P* < 0.001 versus vehicle-treated *db/db* mouse values.

**Figure 9:**
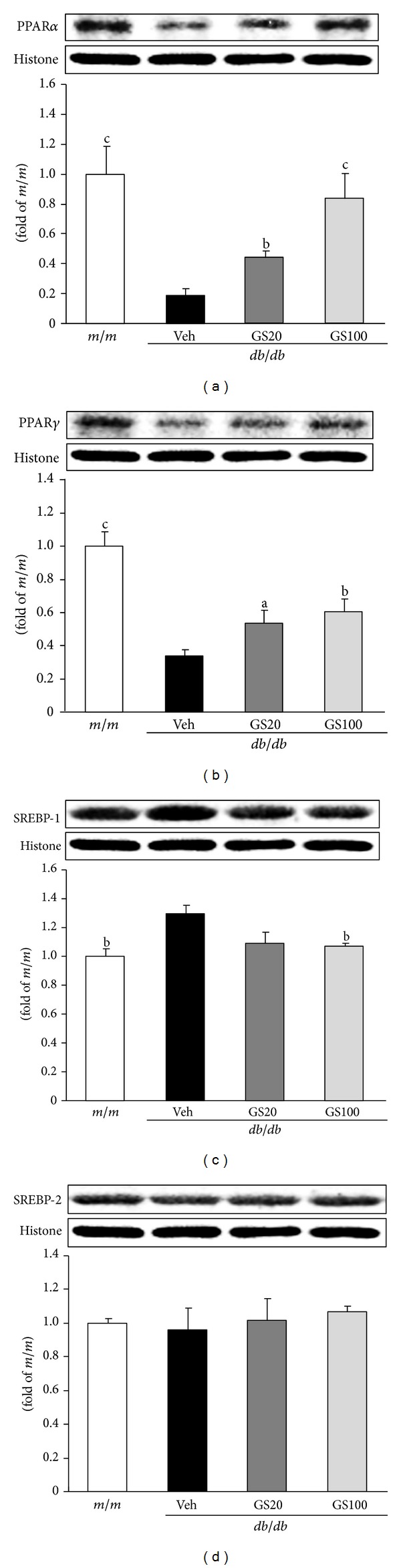
PPAR*α* (a), PPAR*γ* (b), SREBP-1 (c), and SREBP-2 (d) protein expressions in the adipose tissue. *m/m, *Misty; Veh, vehicle-treated *db/db* mice; GS20, GS 20 mg/kg body weight-treated* db/db* mice; GS100, GS 100 mg/kg body weight-treated* db/db* mice. The results are presented as the means ± S.E.M. ^a^ *P* < 0.05, ^b^ *P* < 0.01, ^c^ *P* < 0.001 versus vehicle-treated *db/db* mouse values.

The major biochemical alterations in diabetes are hyperglycemia and dyslipidemia, leading to gluco- and lipotoxicity, which directly or indirectly account for diabetic complications in various organs [59–62]. Longitudinal hyperlipidemia, which is associated with the abnormal expression of transcriptional factors such as peroxisome proliferator activated receptor (PPAR) *α* or sterol regulatory element binding proteins (SREBPs) in the nucleus, increases nonesterified fatty acids (NEFA) uptake and accumulations of triglycerides and cholesterol in tissues. Critical toxicity caused by dyslipidemia is also oxidative stress due to impaired antioxidant defense systems and increased ROS generated by the mitochondrial respiratory chain reaction and glucose autoxidation [63–66]. In this study, the concentrations of triglycerides, total cholesterol, NEFA, high-density lipoprotein (HDL) cholesterol, and very low-density lipoprotein (VLDL)/low-density lipoprotein (LDL) cholesterol in the serum, and triglycerides, total cholesterol, and NEFA in the adipose tissue were significantly elevated in *db/db* compared to those in *m/m* mice. The oral administration of GS affected its favorable influences on the lipid profile of serum and adipose tissue (Table 2, Figure 7). Besides its beneficial effects on lipid metabolism, GS administration promoted antioxidant activity. The elevated ROS and TBARS levels in the serum and adipose tissue were ameliorated nearly to those of *m/m* mice (Table 2, Figure 8). On the other hand, the serum adiponectin level increased on GS treatment, which may be correlated with the decreased serum NEFA level (Table 2). Moreover, lipid metabolism-related protein expressions in the adipose tissue were measured. As shown in Figure 9, protein expressions of transcriptional factors related to lipid regulation, PPAR*α* and PPAR*γ*, were lower in vehicle-treated *db/db* than *m/m* mice, but these decreased expressions were significantly elevated by the 20 or 100 mg GS administration. Also, the elevated SREBP-1 expression in vehicle-treated *db/db* mice was recovered nearly to that of *m/m* mice on 100 mg/kg GS treatment, suggesting that GS modified lipid metabolism, especially triglyceride synthesis.

Previously, we proposed that the suppression of inflammation is possibly linked to antidiabetic effects [67], and other studies have reported that type 2 diabetes can occur through mechanisms related to the inflammatory state [68]. As inflammation is considered to be a major factor contributing to type 2 diabetes [68], we examined proinflammatory markers including TNF-*α* and IL-6 in the serum, and found that GS treatment inhibited serum TNF-*α* and IL-6 (Table 2), indicating that the anti-inflammatory properties of GS result in protection against insulin resistance, consistent with a previous report [69], revealing that the suppression of inflammation *via* the modulation of adiponectin, IL-6, and TNF-*α* is an important protective factor against insulin resistance. It was reported that NF-*κ*B results in insulin resistance by activating proinflammatory cytokines like TNF-*α*, IL-6, IL-1*β*, and resistin, which consequently activates the c-Jun N-terminal kinase (JNK) and NF-*κ*B pathways to create a vicious cycle that exacerbates tissue damage [70].

**Figure 10:**
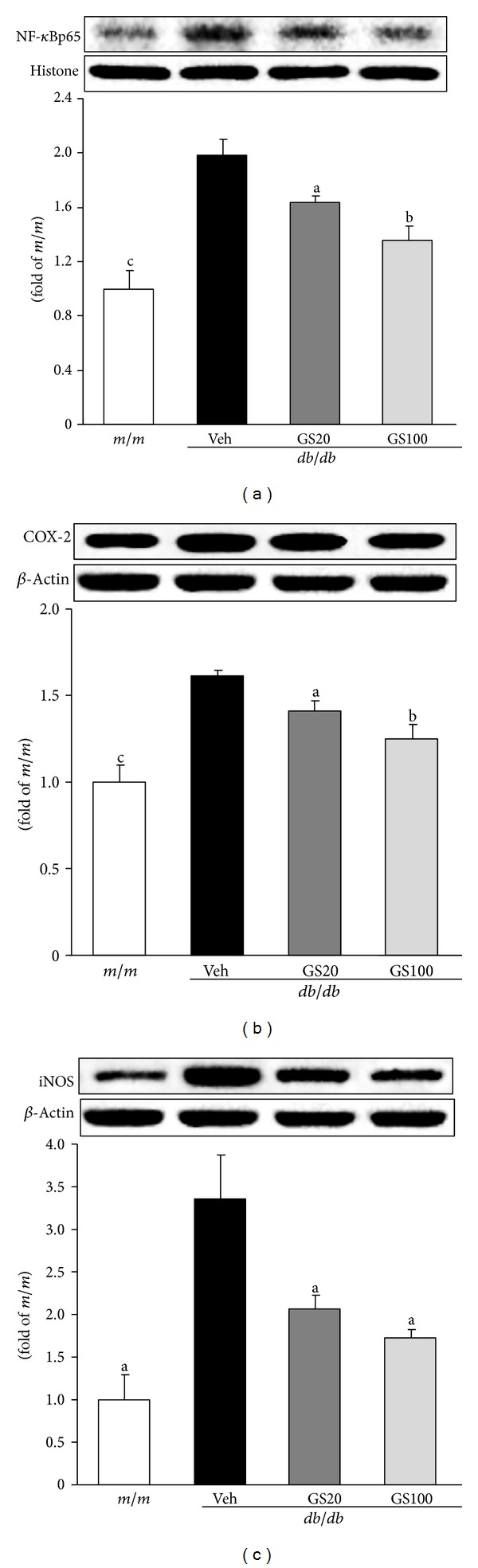
NF-*κ*Bp65 (a), COX-2 (b), and iNOS (c) protein expressions in the adipose tissue. *m/m, *Misty; Veh, vehicle-treated *db/db* mice; GS20, GS 20 mg/kg body weight-treated* db/db* mice; GS100, GS 100 mg/kg body weight-treated* db/db* mice. The results are presented as the means ± S.E.M. ^a^ *P* < 0.05, ^b^ *P* < 0.01, ^c^ *P* < 0.001 versus vehicle-treated *db/db* mouse values.

**Figure 11:**
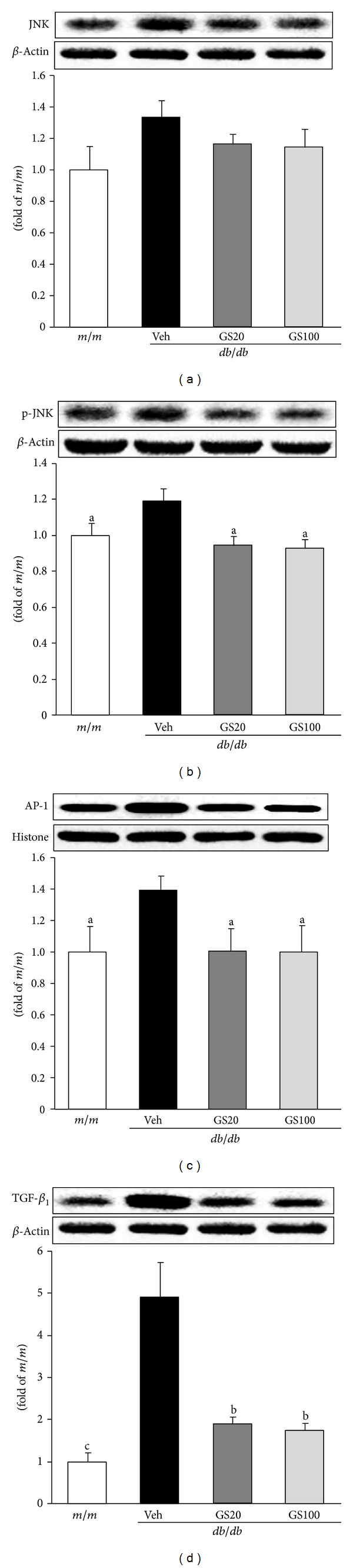
JNK (a), p-JNK (b), AP-1 (c), and TGF-*β*_1_ (d) protein expressions in the adipose tissue. *m/m, *Misty; Veh, vehicle-treated *db/db* mice; GS20, GS 20 mg/kg body weight-treated* db/db* mice; GS100, GS 100 mg/kg body weight-treated* db/db* mice. The results are presented as the means ± S.E.M. ^a^ *P* < 0.05, ^b^ *P* < 0.01, ^c^ *P* < 0.001 versus vehicle-treated *db/db* mouse values.

We further examined proinflammatory NF-*κ*Bp65, COX-2, and iNOS protein levels in the adipose tissue of *db/db* mice, and found that GS treatment downregulated levels of these proteins (Figure 10), suggesting that GS treatment had antidiabetic effects due to its anti-inflammatory actions. These results showing the amelioration of proinflammatory markers, that is, NF-*κ*Bp65 and COX-2 protein expressions, are in parallel with a recent report showing enhanced iNOS protein expression due to NF-*κ*B activation [71]. In addition, it has also been shown that polyphenolic compounds can modulate inflammatory responses *via* the inhibition of COX-2 protein expression through the suppression of JNK activation and inhibition of proinflammatory mediators, like TNF-*α*, by the attenuation of NF-*κ*B and JNK pathways [72]. GS modulated the activation of JNK pathway (JNK→phosphor (p)-JNK→activator protein (AP)-1→TGF-*β*_1_) (Figure 11). These data are consistent with a previous report [73] showing that not only the modulation of oxidative stress and consequent activation of the JNK pathway, but also the suppression of inflammation are involved in the development of dysfunction found in adipose tissue in the presence of diabetes, which, therefore, would make these useful therapeutic targets against adipose tissue in diabetes.

One of our significant findings in this study was GS's suppression of diverse proinflammatory cytokines such as TNF-*α*, IL-6, resistin, and TGF-*β*_1_ that activate the JNK and NF-*κ*B pathways and proinflammatory COX-2 protein expression (Table 2, Figures 10 and 11). In particular, our data showing the suppression of both oxidative stress and inflammation by GS treatment are consistent with our previous report [67], revealing a close relationship between antioxidative and anti-inflammatory actions in diabetes. Thus, based on the results from both our previous and current studies, we suggest a possible mechanism by which the antidiabetic action of GS mediates type 2 diabetes through its dual suppression of oxidative stress and inflammation, as shown in our experiments with *db/db* mice. Consecutively, GS could reduce the increased level of TGF-*β*_1_ in the adipose tissue, showing a reduction in fibrosis. These findings suggest that the hyperglycemic control of GS may, at least in part, be derived from the amelioration of disorders such as fibrosis in adipose tissue.

Although the mechanistic details of GS need to be clarified in future studies, our findings support the therapeutic evidence for GS ameliorating the development of diabetic damage in adipose tissue. An important mechanism of GS's antidiabetic effect is its capacity to lower oxidative stress by reducing ROS generation and lipid peroxidation in adipose tissue. Our data further suggest that another critical mechanism of GS's antidiabetic property is its ability to ameliorate inflammation and fibrosis through modulation of the serum TNF-*α* and IL-6 levels, and oxidative-, inflammation-, and fibrosis-related protein expressions.

## 5. Conclusion

For patients with type 2 diabetes, hyperlipidemia, and insulin resistance, thiazolidinediones and fibrate drugs, both of which activate PPARs, have been widely used [74], but side effects such as body weight gain with an excess increase of the fat mass have been reported in diabetes patients [75]. Alternatively, traditional Chinese medicines with negligible toxic and/or side effects have been used in East Asia, and, among them, medicines containing Corni Fructus as the main ingredient have been used to treat diabetes. Among the bioactive compounds of Corni Fructus, there is therapeutic evidence for GS ameliorating the development of diabetic damage in the serum, kidney, and adipose tissues. In conclusion, GS, a bioactive compound of Corni Fructus, ameliorates the development of diabetic damage in the serum, kidney, and adipose tissues.

## Figures and Tables

**Table 1:** Glucose, leptin, insulin, and C-peptide in serum.

Group	Dose (mg/kg body weight/day)	Glucose (mg/dL)	Leptin (ng/dL)	Insulin (ng/mL)	C-peptide (pg/mL)
*m*/*m*	—	186 ± 25^c^	2.30 ± 0.32^c^	1.82 ± 0.06^b^	177 ± 15^c^
*db*/*db*					
Veh	—	791 ± 42	20.24 ± 0.29	3.72 ± 0.45	1,983 ± 277
GS	20	745 ± 31	18.51 ± 0.75	2.68 ± 0.11^a^	1,135 ± 139^a^
GS	100	683 ± 41	17.57 ± 0.87^a^	2.40 ± 0.04^b^	970 ± 142^b^

*m*/*m*, Misty; Veh, vehicle-treated *db*/*db* mice; GS20, GS20 mg/kg body weight-treated *db*/*db* mice; GS100, GS100 mg/kg body weight-treated *db*/*db* mice. The results are presented as the means ± S.E.M. ^a^ *P* < 0.05, ^b^ *P* < 0.01, ^c^ *P* < 0.001 versus vehicle-treated *db*/*db* mouse values.

**Table 2:** Biomarkers associated with lipids, oxidative stress, and inflammation in serum.

Group	Dose (mg/kg body weight/day)	Triglycerides (mg/dL)	Total cholesterol (mg/dL)	NEFA (mEq/L)	HDL-C (mg/dL)	LDL/VLDL-C (mg/dL)	
*m*/*m*	—	114 ± 9^c^	110 ± 8^c^	0.62 ± 0.02^c^	51.01 ± 3.55^c^	522 ± 4^b^	
*db*/*db*							
Veh	—	263 ± 21	186 ± 8	2.55 ± 0.06	80.95 ± 2.49	570 ± 17	
GS	20	198 ± 16^a^	179 ± 14	1.98 ± 0.04^c^	90.88 ± 7.68	394 ± 21^a^	
GS	100	175 ± 8^b^	163 ± 11	1.56 ± 0.15^c^	95.88 ± 3.16^b^	355 ± 13^c^	

Group	Dose (mg/kg body weight/day)	ROS (fluorescence/min/mL)	TBARS (nmol/mL)	Adiponectin (ng/mL)	Resistin (pg/mL)	TNF-*α* (pg/mL)	IL-6 (pg/mL)

*m*/*m*	—	790 ± 175^a^	18.33 ± 0.46^c^	6.32 ± 0.27^c^	522 ± 4^b^	117 ± 17^a^	11.08 ± 0.33^c^
*db*/*db*							
Veh	—	1,563 ± 144	22.48 ± 0.51	3.18 ± 0.09	570 ± 17	261 ± 28	21.27 ± 2.07
GS	20	950 ± 112^a^	11.88 ± 1.45	3.68 ± 0.12^b^	394 ± 21^c^	162 ± 30^a^	14.90 ± 2.07
GS	100	840 ± 70^b^	9.06 ± 1.19^c^	4.63 ± 0.19^c^	355 ± 13^c^	133 ± 13^b^	12.45 ± 1.19^b^

*m*/*m*, Misty; Veh, vehicle-treated *db*/*db* mice; GS20, GS20 mg/kg body weight-treated *db*/*db* mice; GS100, GS100 mg/kg body weight-treated *db*/*db* mice. The results are presented as the means ± S.E.M. ^a^ *P* < 0.05, ^b^ *P* < 0.01, ^c^ *P* < 0.001 versus vehicle-treated *db*/*db* mouse values.
